# Effect of environmental, economic and health factors on CoVid-19 transmission

**DOI:** 10.6026/97320630017037

**Published:** 2021-01-31

**Authors:** Sobia Anam, Nisar Ahmed Shar

**Affiliations:** 1 National Center in Big Data & Cloud Computing, NED University of Engineering & Technology, Karachi, Pakistan

**Keywords:** CoVid-19 cases per million population, GDP, asthma, humidity, multiple regression, temperature, SPSS, pearson correlation

## Abstract

The severe acute respiratory syndrome (SARS) is affected by meteorological parameters such as temperature and humidity. It is also observed that people having asthma are at risk for SARS. Therefore, it is of interest to report the effect of environmental, economic, and health factors on the spread of CoVid-19. We used data reporting CoVid-19 cases from 24 cities in eight different countries for this analysis. Data was analyzed using multiple linear regressions between these parameters. Data shows that temperature has effects on CoVid-19. A one-degree rise in temperature causes a -0.19 decrease in CoVid-19 cases per million people (log natural value per million populations). The effect of humidity is not significant at a p value of 0.26. Moreover, one-unit increase in asthma and GDP cases per million people show 0.06 and 0.46 increases in CoVid-19 cases, respectively.

## Background

The Severe Acute Respiratory Syndrome Coronavirus 2 (SARS-CoV-2) caused CoVid-19 during the late 2019 in China [1]. Data shows that the virus affects millions of people with thousands of death throughout the world [2]. Hence, CoVid-19 is a serious public health problem worldwide [3]. Data shows that SARS gradually declined with the onset of warm weather during July 2020 [4]. A sharp upswing or lessening in the environmental temperature associated with the cold air outbreak led to an escalation of SARS [5]. SARS-CoV-2 and other closely associated corona viruses, such as Ebola and influenza, have associations with meteorological factors [6]. Report shows that low temperature and humidity favor the spread of the Influenza virus [7]. CoVid-19is associated with meteorological factors [8]. Therefore, it is of interest to report the effect of CoVid-19on environmental, economic, and health factors.

## Methodology

### Data collection:

**Figure 1:**
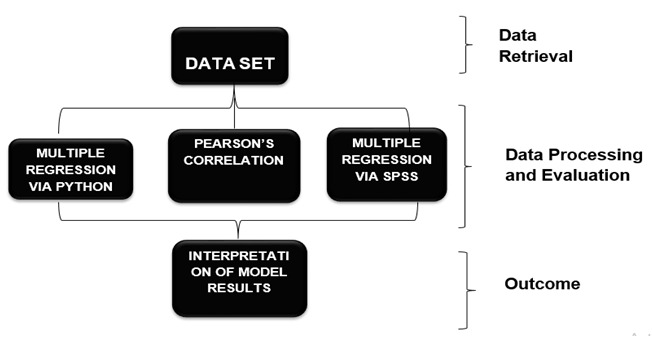
This figure shows the steps that are followed in methodology that data is retrieved and processed via three methods and ledto outcome.

Data from eight countries including 24 major cities and divisions were used in this analysis. (Figure 1 )Table 1 (see PDF) shows cities and total confirmed CoVid-19 cases per million population, temperature, humidity, asthma per million populations, and GDP per million populations (in log natural form) as on July 2020. This data is downloaded from statista, WHO, Delhi state health bulletin, the Newyork times, development health republic of South Africa, Pretoria, Worldmeter and Chicago Website, Larkana coronavirus cases update and the express tribune. Data on temperature and humidity was taken from the weather channel, weather atlas, holiday weather.com weather.com, climate-data.org, accuweather, climates to travel-world climate guide, timeanddate.com, holiday weather.com and holiday spark. Data on asthma cases were taken from statista and the global asthma reports 2018 asthma. The prevalence and cost of illness were taken from the Stock, Karger, the Lancet and the British lung foundation. GDP values have taken from economy of the state of New York, CEIC, government of finance in Pakistan and organization for economic corporation and development.

### Statistical analysis:

Pearson correlation coefficient:

We considered as CoVid-19 cases as dependent factor while temperature, humidity, Asthma and GDP as independent factors for data in Table 1( see PDF).

### Multiple linear regressions:

We used four independent variables like temperature, humidity, asthma, and GDP while, one dependent variable as CoVid-19cases per million populations in log natural form. To analyze the relationship between multiple explanatory variables we used multiple regressions. We used 30% of the data for testing and the remaining 70% for training and 0 random state variables. For training the linear regression model we used X_train and Y_train. To predict the output Y, X_test is used. We analyzed prime coefficients to find the impact on the output Y. Later we predicted the output and compared the actual and predicted values also plotted it. We further analyzed the value of R2 and Root Mean Square Error (RMSE).

### SPSS statistics:

Multiple regressions are used to measure the association of two control measures (Temperature, Humidity) for CoVid-19 cases prediction. Normal probability was plotted to show that every variable in the regression model is normally distributed, and free from univariate outliers. An assessment of the normal probability plot of standardized residuals as well as the scatterplot of standardized residuals against standardized predicted values represented that the assumptions of homoscedasticity, normality, and linearity of residuals were met. Mahalanobis distance her showed that multivariate outliers were of not singnificance (Table 3 - see PDF). Fourth, relatively elevation of tolerances for both predictors (e.g. 0.93) in the regression model showed that multiple linearity would not affect the ability for interpretation of the outcome of the regression model (Table 2 - see PDF).

## Results and Discussion:

Data shows that the virus affects millions of people with thousands of death throughout the world [2]. Hence, CoVid-19 is a serious public health problem worldwide [3]. The severe acute respiratory syndrome (SARS) is affected by meteorological parameters such as temperature and humidity. It is also observed that people having asthma are at risk for SARS. Therefore, it is of interest to report the effect of environmental, economic, and health factors on the spread of CoVid-19.

The normal probability plot (Figure 5 -Figure 6) indicates that each variable in the regression is normally distributed and it is free from univariate outliers. An analysis of the normal probability plot for standardized residuals and the scatter plot for standardized residuals vs. standardized predicted values indicated that the assumptions of normality, linearity and homoscedasticity (meaning same variance) of residuals were met. Temperature and humidity accounted for 66% of the variability in CoVid19 cases with R^2^ = 0.66, adjusted R^2^= 0.39, F (2, 21) = 8.25, p <001 (Table 4 and Table 5 - see PDF). This shows that humidity does not have a significant impact on CoVid-19ccases. However, temperature has a major impact on CoVid-19 cases. The beta value of temperature (beta = - 0.21, p < 0.001) indicates that if temperature increases by 1 degree Celsius, 0.21 will decrease CoVid19 cases. Therefore, the null hypothesis that temperature does not have a significant impact on CoVid19 cases is rejected. It can be concluded that temperature has a significant impact on CoVid19 affected people. But we failed to reject the null hypothesis that humidity does not have a significant impact on CoVid19 affected people.

A simple regression with temperature as a independent variable shows that temperature accounted for 64% of the variability in CoVid19 cases with R2 = 0.64, adjusted R2 = 0.38, F(1, 22) = 14.96, p < 001 (Table 6 and Table 7 - see PDF). We obtained data by taking 70% as training data and 30% as testing data from Table 1( see PDF) by keeping random state variable as 0. The intercept was 9.1 and the Root Mean Square Error was 1.53 with R2 = 0.641. The value of temperature is 18 degrees Celsius, humidity is 78 %, asthma cases as 1.97 and GDP as 11.29 per million populations with natural log was used for the validation. The model predicted 8.67 CoVid-19 cases per million populations in log natural form. It should be noted that the actual value is 9.9 CoVid-19 cases per million populations in natural log form. Hence, it showed that model have almost 64% accuracy (Figure 7-Figure 8).

**Figure 2:**
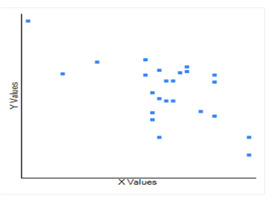
The correlation between the temperature (X) in Celsius and CoVid-19case per million population with log natural (Y), and it infers that a moderate negative correlation, which means there is a tendency for high X values to go with low Y values and vice versa.

Data shows that a one degree Celsius rise in temperature will reduce the CoVid-19 cases by -0.19 times as analyzed using the python tool described in this article in Figure 9. We trained the model with data in Table 1 (see PDF). We found that same results were obtained using the SPSS tool where we found that the beta value of temperature (beta = - 0.21, p < 0.001) indicates that if temperature increases by one-degree Celsius, CoVid-19 cases will be decreased by 0.21. We further used the Pearson correlation between temperature and CoVid19 where a moderate negative correlation between CoVid-19 cases and average temperature is recorded (Figure 2). Thus, these data strongly support that there is a negative relationship between temperature and CoVid-19.

**Figure 3:**
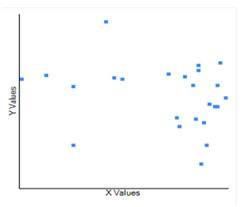
The correlation between the Humidity (X) and CoVid-19case per million populations with log natural (Y), which infers that technically it is a negative correlation, the relationship between X and Y- is only weak.

**Figure 4:**
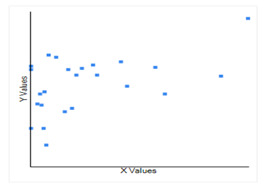
The correlation between the Asthma cases per million populations (X) and CoVid-19case per million population with log natural (Y), it infers that technically it is a positive correlation, the relationship between X and Y cases is weak is only weak.

**Figure 5:**
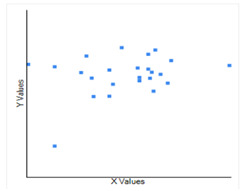
The correlation between the GDP per million populations with log natural (X) and CoVid-19case per million populations with log natural (Y), it concludes that technically it is a positive correlation, the relationship between X and Y is weak.

**Figure 6:**
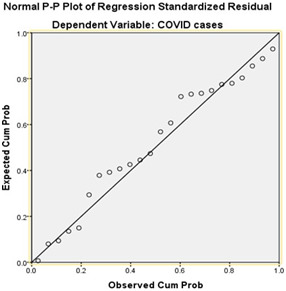
Normal P-P plot of Regression Standardized Residual Dependent variable CoVid-19cases, normal probability plot is showing that every variable in the regression model is normally distributed, and free from univariate outliers.

**Figure 7:**
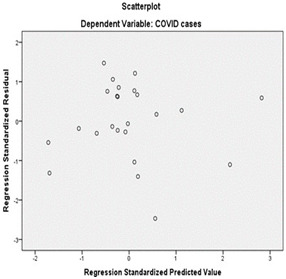
Scatter plot of standardized residuals against standardized predicted values represented that the assumptions of homo scedasticity, normality, and linearity of residuals were met.

**Figure 8:**
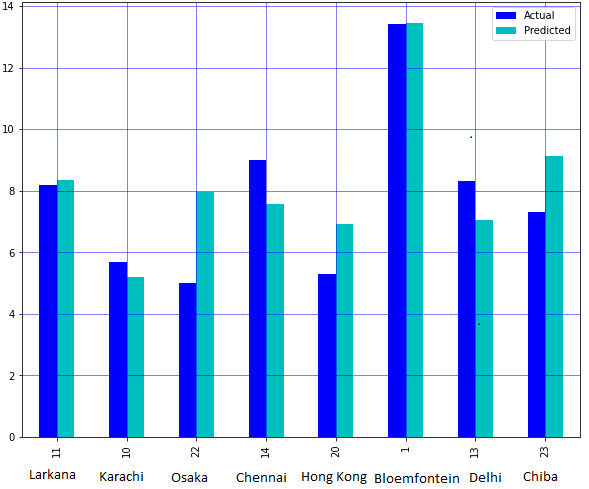
The association between actual and predicted value of CoVid-19cases per million populations (in log natural) per city.

**Figure 9:**
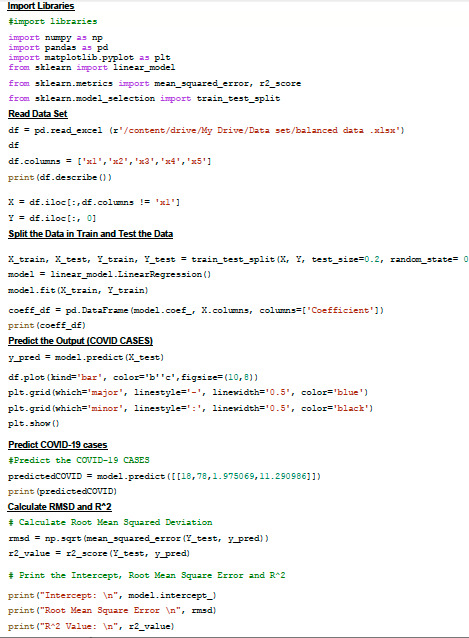
The given python code is used for multiple regressions.

We used four main features for this study. The second feature used was humidity. Data shows that if 1% of humidity increases the number of CoVid-19 cases per million populations. This will decrease with -0.03 times as the coefficient value shows a slight reduction of CoVid-19cases. Humidity does not have any significant impact on CoVid-19 with a p value of 0. 26. The Pearson correlation value R is negative with -0.2415 showing no strong relationship (Figure 3). Therefore, humidity does affect the CoVid19 with no strong impact. Asthma does not have such a strong association with CoVid-19. A one-unit increase in asthma cases occurs per million populations. Hence, the cases of CoVid-19 will increase by only 0.057 with a Pearson correlation value R is 0.49. Thus, asthma has not a strong association with CoVid-19. The coefficient value is 0.46 for GDP with CoVid-19 (Figure 4). Therefore, GDP and CoVid-19 does not show considerable relation.

## Conclusion

Data shows that temperature has effects on CoVid-19. A one-degree rise in temperature causes a -0.19 decrease in CoVid-19 cases per million people (log natural value per million populations). The effect of humidity is not significant at a p-value of 0.26. Data also shows that one-unit increase in asthma and GDP cases per million people resulted in 0.057 and 0.46 increase, respectively in CoVid-19 cases.
